# Depressive, anxiety symptoms and their co-occurrence among women seeking antenatal care in Bangladesh

**DOI:** 10.1038/s41598-025-01801-w

**Published:** 2025-05-17

**Authors:** Md Hafizur Rahman, Ridwana Maher Manna, Nasimul Ghani Usmani, Pradip Chandra, Md. Abdullah Al Mamun, Md Robed Amin, Maruf Ahmed Khan, Helal Uddin Ahmed, S. M. Hasibul Islam, Md. Shariful Islam, Tasnu Ara, Ema Akter, Anisuddin Ahmed, Mohammad Sohel Shomik, Shams El Arifeen, Aniqa Tasnim Hossain, Ahmed Ehsanur Rahman

**Affiliations:** 1 Maternal and Child Health Division, International Centre for Diarrhoeal Disease Research, Bangladesh (icddr,b), 68 Shaheed Tajuddin Ahmed Ave, Mohakhali, Dhaka, 1212 Bangladesh; 2 Non-communicable Disease Control (NCDC), Director General of Health Services (DGHS), Ministry of Health and Family Welfare (MoHFW), Mohakhali, Dhaka, 1212 Bangladesh; 3 Department of Child, Adolescent, and Family Psychiatry, National Institute of Mental Health (NIMH), Sher-e-Bangla Nagar, Dhaka, 1207 Bangladesh

**Keywords:** Antenatal, Pregnancy, Mental disorders, Depression, Anxiety, Outpatients, Bangladesh, Psychology, Medical research, Risk factors

## Abstract

Anxiety and depression are significant concerns among antenatal women in Bangladesh. Despite the critical need for tailored mental health care in health facilities, studies on these symptoms in this demographic remain lacking. Therefore, our study aimed to assess the levels, distribution, and associated factors of depressive and anxiety symptoms and their co-occurrence among women seeking antenatal care at a public healthcare facility in Bangladesh. We conducted a cross-sectional study between May 2024, and June 2024, among women seeking antenatal care (ANC) care in Durgapur Upazila Health Complex, a primary-level public health facility in Bangladesh. Among 640 women who received ANC care, 638 participated in the study. Depressive symptoms were assessed by the Patient Health Questionnaire-9 (PHQ-9), and anxiety symptoms were assessed by the Generalized Anxiety Disorder-7 (GAD-7). Bivariate and multivariable logistic regression were conducted to determine factors contributing to depressive and anxiety symptoms. About 39% of participants had depressive symptoms and 50% had anxiety symptoms, with 26% experiencing both simultaneously. No participants had severe overall depressive or anxiety symptoms. PHQ-9 data indicated half experienced daily fatigue, while GAD-7 data showed over half experienced daily nervousness and two-fifths had daily fears. Women in the second and third trimesters had 43% (aOR: 0.57, 95% CI: 0.36–0.89) and 58% (aOR: 0.42, 95% CI: 0.24–0.71) lower odds of depressive symptoms compared to those in the first trimester respectively. Women with 11 years or more education had 40% (aOR: 0.60, 95% CI: 0.38–0.94) lower odds of anxiety. Additionally, women in the second and third trimesters had 40% (aOR: 0.60, 95% CI: 0.37–0.97) and 49% (aOR: 0.59, 95% CI: 0.29–0.91) lower likelihood of co-occurrence compared to those in the first trimester and women with 6–10 years of education had 48% (aOR: 0.52, 95% CI: 0.34–0.79) and those with 11 or more years had 52% (aOR: 0.48, 95% CI: 0.29–0.81) lower likelihood of co-occurring depressive and anxiety symptoms compared to women with 5 years or less education. Our study found a high prevalence of depressive and anxiety symptoms among antenatal care seekers, with notable co-occurrence of these conditions. Given these findings, there is an urgent need for targeted mental health support for these women, especially those in their first trimester and those with limited education.

**Supplementary Information:**

The online version contains supplementary material available at 10.1038/s41598-025-01801-w.

## Introduction

The Global Burden of Disease Study 2019 highlighted that women experience higher rates of depressive and anxiety symptoms compared to men, with these symptoms becoming more prevalent over the years^1^. Pregnant women, in particular, are more susceptible to depressive and anxiety symptoms due to the significant psychological changes that occur during pregnancy^2^. According to the World Health Organization (WHO), approximately 10% of women worldwide experience some form of mental health condition during pregnancy^3^. In Bangladesh, the burden of mental health disorders, especially depression and anxiety, is similarly high among pregnant women^4–9^.

The impact of maternal depressive and anxiety symptoms during pregnancy is associated with increased risks of pre-eclampsia, hemorrhage, impaired intrauterine growth, cesarean sections, and even stillbirths^10,11^. Alarmingly, suicide, one of the leading causes of maternal death during pregnancy, is also linked to severe mental disorders, with suicidal ideation often continuing into the postpartum period, contributing to maternal mortality^12,13^. Several systematic studies reported that the consequences of maternal mental disorders are not confined to the mother alone. Adverse health outcomes were found among newborns and child health, such as higher rates of early child mortality, preterm birth, low birth weight, spontaneous abortion, small for gestational age along with admissions to the neonatal intensive care unit. Additionally, poor nutritional status, delayed motor, cognitive, and language development, and behavioral problems have also been significantly associated^14–17^.

In Bangladesh, mental health concerns are becoming a growing crisis. The country’s mental healthcare system faces significant obstacles, including a shortage of public facilities, insufficient trained professionals, underfunding, and widespread societal stigma^18^. With just 50 outpatient mental healthcare facilities serving a population of 165 million, primarily located in urban areas, the gap in mental healthcare access is glaring^18^. Additionally, there are only 1.17 mental health workers per 100,000 people, most of whom are based in tertiary care centers in major cities^19^. The situation has been further worsened by the COVID-19 pandemic and climate-related stressors, heightening the demand for accessible mental healthcare in the country^18,20,21^.

Given the high prevalence of depressive and anxiety symptoms, coupled with limited access to mental healthcare and a shortage of providers in Bangladesh, the government has prioritized the establishment of facility-based tele-mental health services, such as well-being centers, across all levels of healthcare by 2030^22,23^. In this model, patients seeking outpatient care, including antenatal women at antenatal care (ANC) outdoors, will be identified and referred to tele-mental health services as needed.

Between 2009 and 2022, several studies in Bangladesh examined depressive and anxiety symptoms among antenatal women: however, most focused solely on depression. Some investigated depressive symptoms in relation to gestational diabetes, pregnancy stages, suicidal ideation, or sleep quality, without assessing anxiety^4–7^. One study explored anxiety symptoms alone, while another examined maternal mental health disorders with low birth weight^9,24^. Only a single study assessed both conditions together, but it was conducted in rural community settings rather than healthcare facilities^8^. Although the 2022 Bangladesh Demographic and Health Survey included data on depressive and anxiety symptoms among ever-married women of reproductive age, it did not specifically report the burden and symptom patterns among antenatal women, particularly those seeking facility-based care^25^. Given Bangladesh’s low postnatal care utilization, health facilities often engage primarily with antenatal care seekers^26^. Understanding mental health disorders in this group is critical for developing and providing health facility-based interventions tailored to the need. However, to date, no study has explored the levels, distribution, and co-occurrence of depressive and anxiety symptoms among antenatal care seekers in Bangladesh. Therefore, our study aimed to assess the levels, distribution, and associated factors of depressive and anxiety symptoms and their cooccurrence among women seeking antenatal care at a public healthcare facility in Bangladesh.

## Method

### Study design and setting

We conducted a cross-sectional survey among pregnant women in the antenatal period who sought and received ANC at the Durgapur Upazila Health Complex, a primary-level public health facility located in the Netrokona district of Mymensingh Division, Bangladesh. Durgapur Upazila, situated in the northern part of Bangladesh along the border with India, has a population of 242,445, with 119,372 males and 123,073 females^27^. The geographical location of Durgapur Upazila is provided in supplementary material 1.

### Sampling, sample, and data collection

The study employed a consecutive sampling method to recruit participants. Data collection took place between May 2024, and June 2024. During this period, all women who visited the hospital for antenatal care (ANC) and were registered in the ANC register book were invited to participate in the study. A total of 640 women were registered in the ANC register book during the data collection period and received ANC services at the hospital. Among these, two women declined to participate, resulting in a final sample of 638 women who consented to participate in the survey, yielding a response rate of 99.7%.

### Procedure

Participants were interviewed on the day of their ANC visit and confidentiality during the interview was maintained. Each participant was assigned a unique ID number corresponding to the ANC register book. Women who returned for subsequent ANC visits during the study period were not re-interviewed if they had already participated. Data collection was conducted using a structured questionnaire, which was digitized and administered via tablets. Two trained data collectors carried out the interviews. The questionnaire was designed to capture information on depressive and anxiety symptoms, as well as socio-demographic, economic, and pregnancy-related factors.

A pilot test was conducted with 10 respondents to identify any issues with the data collection tools and software. Based on the pilot test results, necessary adjustments were made to the questionnaire and software. The data gathered during the pilot test were excluded from the main study analysis. Each day, data collectors manually checked the collected data for completeness and consistency before syncing it on the tablet to send to the field research supervisor. A quality check was performed on 5% of the daily collected data by the field research supervisor, and any inconsistencies were promptly addressed.

### Data collection tools and categorization

The socio-demographic and economic variables encompassed age in years, years of education (0–5 years, 6–10 years & >= 11 years), and household income (< 10000, Very low; 10000–19999, Lower middle; 20000–29999, Middle; >=30000, High) in taka per month. Age, education, and household income data were initially collected as continuous variables and were later categorized for analysis. Age groups were classified as 17–20, 21–25, and 26–40 years. The pregnancy-related variables encompassed total gestational weeks, total number of living children (No children, 1, ≥ 2), whether it is first pregnancy (yes, no), mode of last delivery (normal, C-section, abortion), history of miscarriage (yes, no), history of dead child (yes, no), and have chronic disease for 12 months or more (yes, no), and total number of chronic disease (1, ≥ 2). A detailed description of the explanatory variables is given in supplementary materials 2.

We assessed depressive symptoms using the Patient Health Questionnaire-9 (PHQ-9), a widely used screening tool consisting of nine items on a 4-point Likert scale. The PHQ-9 items include: (1) Anhedonia; (2) Depressed mood; (3) Sleep problems; (4) Low energy; (5) Appetite change; (6) Low self-esteem; (7) Concentration difficulties; (8) Retardation; (9) Suicidal ideation^28^. The PHQ-9, adapted and validated for Bengali-speaking populations, demonstrated cultural relevance (Cronbach’s alpha 0.837) and has been extensively used in Bangladesh^21,29^. Additionally, we assessed anxiety symptoms using the Generalized Anxiety Disorder-7 (GAD-7), a seven-item anxiety screening scale on a 4-point Likert scale. The GAD-7 items are: (1) Nervousness; (2) Unable to control worry; (3) Worrying; (4) Trouble relaxing; (5) Restlessness; (6) Irritability; (7) Fear of awful events^30^. The GAD-7, adapted for local use (Cronbach’s alpha 0.869), has been employed in both clinical and non-clinical contexts in Bangladesh^21,31^. Both PHQ-9 and GAD-7 utilized a recall period of two weeks.

To establish the presence of depressive and anxiety symptoms, we utilized a clinical cut-off score of 10 for both PHQ-9 and GAD-7, in line with established best practices^32,33^, validations in South Asian countries^34^, and previous studies in Bangladesh^21^. A meta-analysis involving 6,725 participants from 29 studies indicated that the PHQ-9 achieved maximum sensitivity (0.88) and specificity (0.85) at a cutoff score of 10, making it relevant for research^35^. Similarly, a meta-analysis involving 5223 participants in 11 studies showed that the GAD-7 had optimized sensitivity (0.83) and specificity (0.84) at the cutoff score of 10^36^. The PHQ-9 and GAD-7 scales were categorized into ‘none’ (0–4), ‘mild’^5–9^, ‘moderate’^10–14^, ‘moderately severe’^15–19^, and ‘severe’ (20–27 for PHQ-9 and 15–21 for GAD-7) based on established scoring cut-offs for each symptom severity level. If a participant was identified as having both depressive and anxiety symptoms, we recorded it as the co-occurrence of depressive and anxiety symptoms in binary response format.

### Statistical analysis

Statistical analysis was performed using Stata/MP 17.0. Any missing values in the dependent variables were excluded from the analysis. Descriptive statistics were generated for sociodemographic and pregnancy-related variables, and binary logistic regression was employed to examine the associations between independent variables and the dependent variables. The associations between depressive symptoms, anxiety symptoms, and their co-occurrence with covariates were examined by both bivariate and multivariable logistic regression analyses. Variables with a Variance Inflation Factor (VIF) below 2, indicating no multicollinearity, were included in the multivariate logistic regression analysis (supplementary material 3)^37^. Furthermore, for the multivariate logistic regression models, the goodness of fit was evaluated using the Hosmer-Lemeshow test, with a p-value greater than 0.05 indicating a good fit. Models that did not meet this criterion were excluded from the final analysis. A detailed description of the models excluded is described in supplementary material 4.

### Ethical consideration

Research and ethical approval for the study were obtained from the Institutional Review Board (IRB) of the International Centre for Diarrhoeal Disease Research, Bangladesh (icddr, b) (PR-22103). Study protocols were diligently followed in the conduct of this research to ensure compliance with all relevant guidelines and regulations. Participants were thoroughly informed about the study and its objectives, and written consent was obtained before the interviews. All identifying information was removed before analysis to ensure anonymity. Privacy and confidentiality were rigorously maintained throughout the study.

## Results

Table 1 presents the demographic characteristics and pregnancy information of the participants. The study comprised a total of 638 participants. The majority of participants were young mothers aged 17 to 25 years, completed 6–10 years of education, belonged to lower middle-income households, and were in 13 to 28 weeks gestational age.

**Table 1:** Demographic characteristics and pregnancy information of the participants (*n* = 638).

Characteristics	*n*	%
Age, years		
17–20 years	237	37
21–25 years	232	36
26–40 years	169	26
Education, years		
Education 0–5, years	185	29
Education 6–10, years	307	48
Education ≥ 11, years	146	23
Household income, taka per month		
Very low (< 10000)	9	1
Lower middle (10000–19999)	371	58
Middle (20000–29999)	181	28
High (≥ 30000)	77	12
Gestational period		
1 to 12 weeks	96	15
13 to 28 weeks	393	62
29 to 40 weeks	149	23
Total living children		
No children	279	44
1	232	36
≥ 2	127	20
First pregnancy?		
No	395	62
Yes	243	38
Mode of last delivery, if applicable (*n* = 395)		
Normal delivery	288	73
C-Section	74	19
Abortion	33	8
Miscarriage history		
No	569	89
Yes	69	11
History of child death		
No	599	94
Yes	39	6
Chronic disease		
No	516	81
Yes	122	19
Total number of chronic disease (*n* = 122)		
1	118	97
≥ 2	4	3

**Fig. 1:**
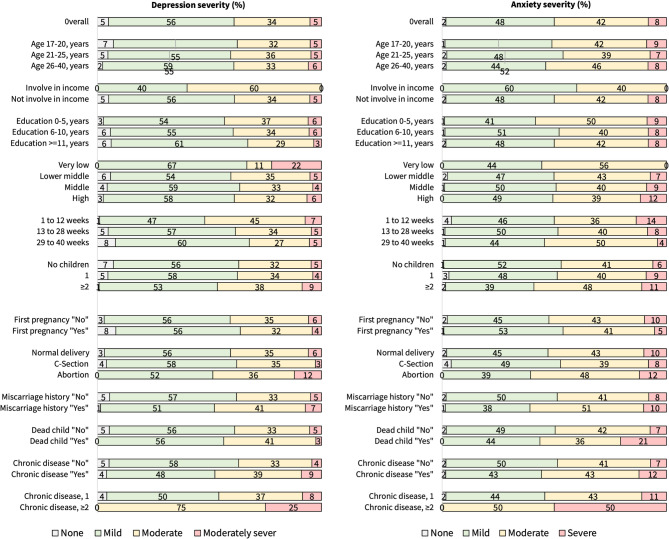
Severity of depressive and anxiety symptoms across demographics and pregnancy condition of the respondents in percentage.

Figure 1 illustrates the severity of depressive and anxiety symptoms across demographics and pregnancy information. Overall, 39% (95% CI: 35–43) had depressive symptoms and 50% (95% CI: 46–54) had anxiety symptoms. In terms of severity, the majority of the depressive and anxiety symptoms were mild (56% for depressive and 48% for anxiety symptoms) to moderate (34% for depressive and 42% for anxiety symptoms).

**Fig. 2:**
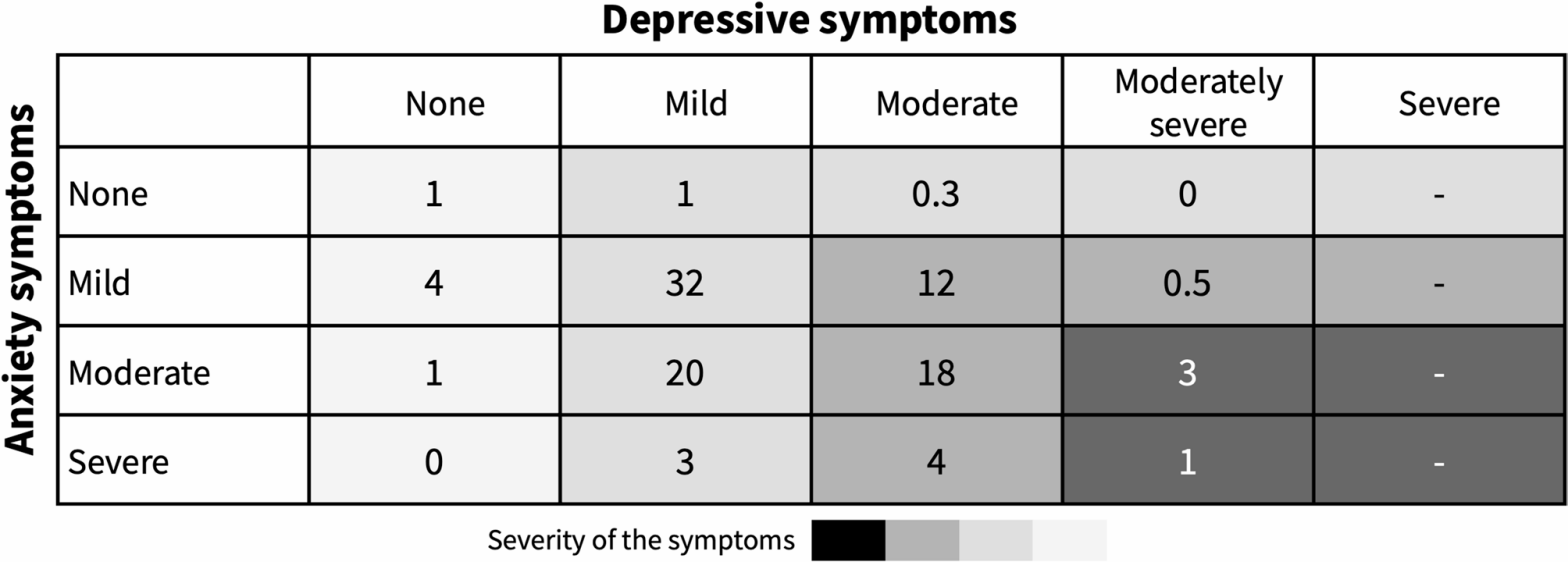
Co-existence and overlap between depression and anxiety at different severity levels in percentage.

Figure 2 illustrates the co-existence and overlap between depressive and anxiety symptoms at different severity levels. Overall, 26% (95% CI: 23–29) had both depressive and anxiety symptoms. None of the participants had severe depressive and severe anxiety symptoms. Around one-fifth of the participants had both moderate depressive and anxiety symptoms.

**Fig. 3:**
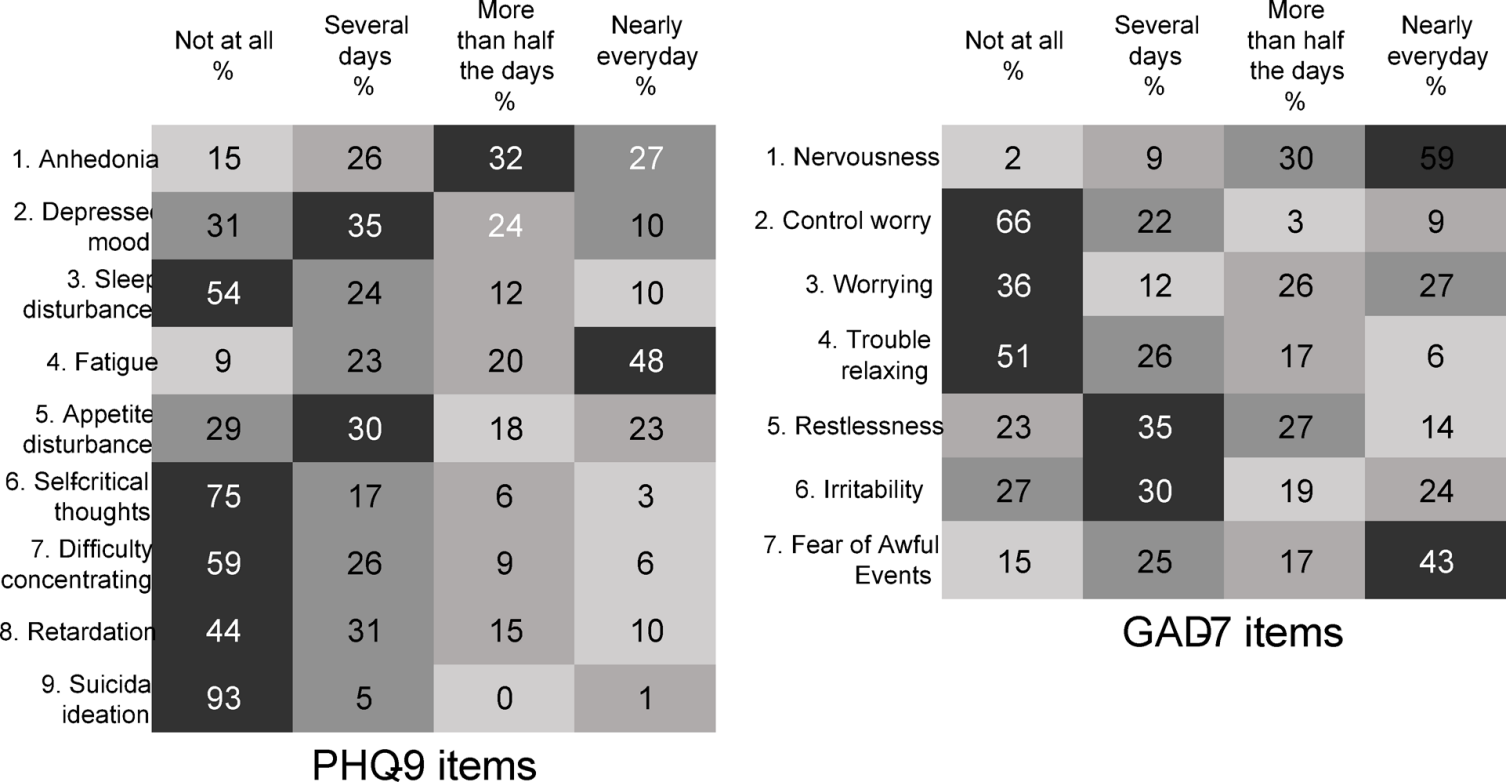
Heatmap of responses to PHQ-9 and GAD-7 items in percentage.

Figure 3 illustrates the percentage response of depressive (PHQ-9) and anxiety (GAD-7) symptoms. The PHQ-9 data revealed that around half of the participants experienced fatigue every day. The GAD-7 data indicated that over half of the participants experienced nervousness and two-fifths were afraid of awful events nearly every day.

Table 2 presents the associations of demographic and pregnancy factors with depressive symptoms. Women in 2nd trimester of the gestational period had 43% (aOR: 0.57, 95% CI: 0.36–0.89) and 3rd trimester of the gestational period had 58% (aOR: 0.42, 95% CI: 0.24–0.71) lower chance of having depressive symptoms compared to women in 1st trimester of gestational period.

**Table 2:** Associations of demographic and pregnancy factors with depressive symptoms.

Characteristics	Bivariable analysis		Multivariable analysis	
	OR (95% CI)	*p*-value	aOR (95% CI)	*p*-value
Education, years				
0–5	Ref		Ref	
6–10	0.85 (0.59–1.24)	0.403	0.89 (0.60–1.31)	0.547
≥11	0.64 (0.41–1.01)	0.055	0.64 (0.40–1.03)	0.068
Gestational period				
1 to 12 weeks	Ref		Ref	
13 to 28 weeks	0.57 (0.37–0.90)	0.015	**0.57 (0.36–0.89)**	**0.015**
29 to 40 weeks	0.44 (0.26–0.74)	0.002	**0.42 (0.24–0.71)**	**0.001**
Total living children				
No children	Ref		Ref	
1	1.03 (0.72–1.47)	0.892	0.94 (0.65–1.36)	0.738
≥ 2	1.48 (0.97–2.27)	0.069	1.29 (0.82–2.01)	0.282
History of miscarriage				
No	Ref		Ref	
Yes	1.50 (0.91–2.47)	0.114	1.14 (0.63–2.04)	0.667
Presence of chronic disease				
No	Ref		Ref	
Yes	1.54 (1.04–2.30)	0.033	1.51 (0.95–2.40)	0.083

Table 3 presents the associations of sociodemographic and pregnancy factors with anxiety symptoms of the study participants. Women with 11 years or more education had 40% (aOR:0.60, 95% CI: 0.38–0.94) lower odds of having anxiety symptoms compared to the women with 5 years or less education.

**Table 3:** Associations of sociodemographic and pregnancy factors with anxiety symptoms.

Characteristics	Bivariable analysis		Multivariable analysis	
	OR (95% CI)	*p*-value	aOR (95% CI)	*p*-value
Education, years				
0–5	Ref		Ref	
6–10	0.66 (0.45–0.95)	0.024	0.69 (0.47–1.01)	0.059
≥11	0.56 (0.36–0.86)	0.009	**0.60 (0.38–0.94)**	**0.026**
Total living children				
No children	Ref		Ref	
1	1.07 (0.76–1.52)	0.693	0.98 (0.69–1.41)	0.928
≥ 2	1.63 (1.07–2.49)	0.024	1.40 (0.90–2.18)	0.141
History of miscarriage				
No	Ref		Ref	
Yes	1.64 (0.98–2.73)	0.058	1.53 (0.85–2.75)	0.154
Presence of chronic disease				
No	Ref		Ref	
Yes	1.33 (0.89–1.98)	0.159	1.12 (0.71–1.77)	0.627

Table 4 presents the associations of sociodemographic and pregnancy factors with co-occurring depressive and anxiety symptoms. Women with 6–10 years of education had 48% (aOR: 0.52, 95% CI: 0.34–0.79) lower likelihood, and women with 11 or more years of education had 52% (aOR: 0.48, 95%CI: 0.29–0.81) lower likelihood of co-occurrence of both depressive and anxiety symptoms compared to women with 5 years or less education. Additionally, women in 2nd trimester had 40% (aOR:0.60, 95% CI: 0.37–0.97) and 3rd trimester had 49% (aOR:0.59, 95% CI: 0.29–0.91) lower likelihood of co-occurring depressive and anxiety symptoms compared to women in 1st trimester.

**Table 4:** Associations of sociodemographic and pregnancy factors with co-occurrence of depressive and anxiety symptoms.

Characteristics	Bivariable analysis		Multivariable analysis	
	OR (95% CI)	p-value	aOR (95% CI)	p-value
Education, years				
0–5	Ref		Ref	
6–10	0.51 (0.34–0.76)	0.001	**0.52 (0.34–0.79)**	**0.002**
>=11	0.48 (0.25–0.79)	0.004	**0.48 (0.29–0.81)**	**0.005**
Gestational period				
1 to 12 weeks	Ref		Ref	
13 to 28 weeks	0.59 (0.37–0.96)	0.032	**0.60 (0.37–0.97)**	**0.038**
29 to 40 weeks	0.54 (0.30–0.94)	0.029	**0.51 (0.29–0.91)**	**0.022**
Total living children				
No children	Ref		Ref	
1	1.03 (0.69–1.55)	0.87	0.88 (0.58–1.34)	0.559
≥ 2	1.64 (1.04–2.60)	0.033	1.24 (0.76–2.01)	0.396
History of miscarriage				
No	Ref		Ref	
Yes	1.78 (1.06–3.01)	0.03	1.49 (0.81–2.78)	0.203
Presence of chronic disease				
No	Ref		Ref	
Yes	1.52 (0.99–2.32)	0.054	1.32 (0.80–2.19)	0.284

## Discussion

To our knowledge, this is the first study to explore the prevalence and co-occurrence of depressive and anxiety symptoms among women seeking antenatal care (ANC) in public healthcare facilities in Bangladesh. Our findings provide new insights into the mental health burden experienced by this population and reveal critical factors associated with these conditions. Our findings reveal a notably high burden of depressive and anxiety symptoms in this population, especially when compared to estimates from other low- and middle-income countries (LMICs)^38^. While prior research has reported varying prevalence rates across different subgroups and settings, our findings underscore a concerning mental health burden specifically among ANC attendees at public facilities^4–7,39^. This elevated prevalence may reflect the compounded stressors these women face—including socioeconomic hardship, limited mental health resources, and overburdened health systems—amplified by the physical and emotional demands of pregnancy. The results suggest a critical gap in routine maternal care and highlight the urgent need for integrated mental health screening and support services within ANC settings to ensure timely identification and intervention.

Similarly, we found that half of the participants experienced anxiety symptoms, an alarmingly high rate that exceeds prior estimates from LMICs^40^ and several rural-based studies in Bangladesh^9,39^. While previous research has documented elevated anxiety levels during pregnancy, especially in underserved areas, the higher prevalence observed in our study may reflect the cumulative stressors faced by women seeking care in resource-constrained public health settings. These include long wait times, limited provider availability, and concerns about the quality and continuity of care. Additionally, socioeconomic stress, fear of complications, and insufficient social support may further heighten anxiety during pregnancy. These findings point to an urgent need for accessible, patient-centered mental health services tailored to the unique realities of antenatal care seekers in public facilities^41,42^.

Our findings also revealed a substantial overlap between depressive and anxiety symptoms among antenatal care seekers, indicating a significant overlap of these mental health conditions among antenatal care seekers. This aligns with previous research showing that depressive and anxiety symptoms frequently co-occur during pregnancy^21^. Studies conducted in rural Bangladesh^8^ and among women with low birth weight offspring^5^ have also found the presence of depressive and anxiety symptoms. The high rate of co-occurrence highlights the critical need for integrated screening and intervention strategies that address both depressive and anxiety symptoms simultaneously. By implementing screening protocols that assess for both depressive and anxiety symptoms, healthcare providers can better identify at-risk women who may otherwise go unnoticed.

Interestingly, only one of the participants in our study had severe anxiety symptoms with moderately severe depressive symptoms. This contrasts with earlier findings in Bangladesh, where a small proportion of women with gestational diabetes or rural community women reported severe depressive symptoms^4^ or suicidal ideation^7^. This discrepancy could be due to differences in the populations studied or as our sample included only those seeking antenatal care in public facilities, potentially excluding more vulnerable women.

Our analysis of symptom-specific data revealed that a large number of women experienced daily fatigue, nervousness, and persistent fear of awful events. These findings are consistent with global research showing that physical and emotional exhaustion is common during pregnancy, often linked to hormonal changes and psychosocial stressors^43^. The high prevalence of fatigue, fear, and nervousness may come from fear of childbirth^43^. This underscores the need for targeted mental health support that includes tailored interventions such as stress management counseling, childbirth education to address fears, and routine mental health screenings integrated into antenatal care to identify and address these symptoms early.

Our study also found that women in the second and third trimesters of pregnancy were less likely to experience depressive symptoms and co-occurrence of depressive and anxiety symptoms compared to those in the first trimester. This finding aligns with a cohort study from Bangladesh, which reported that depressive symptoms tend to decrease as pregnancy progresses^6^. In contrast, another study in Bangladesh reported that antenatal anxiety increases as gestational age increases^9^. This highlights the importance of conducting multiple screenings for depressive and anxiety symptoms throughout pregnancy, as mental health symptoms may fluctuate, with heightened vulnerability during the first trimester due to the stress of adapting to pregnancy and the third trimester due to the anticipation of childbirth and motherhood^44^. However, this pattern might reflect a period of adjustment during the first trimester, when women are first grappling with pregnancy changes and potential uncertainties. By the second and third trimesters, women may adapt better to these changes and experience reduced stress.

Education emerged as a significant protective factor in our study. Women with more years of education had a lower likelihood of experiencing anxiety symptoms and a lower likelihood of co-occurring depressive and anxiety symptoms compared to those with five years or less of education. This aligns with previous findings from Bangladesh, where higher education levels were associated with reduced anxiety symptoms^9^. Education likely enhances women’s coping mechanisms, emotional resilience, and health literacy, enabling them to recognize symptoms and seek timely support for mental health^45^. Furthermore, educated women often have better access to social and economic resources, including stronger support networks and economic stability, which buffer against depressive and anxiety symptoms during pregnancy^46^. Given the significant role of education, targeted Social and Behavioral Change Communication initiatives can be designed to promote self-care practices and adequate rest, addressing the common issue of fatigue during pregnancy.

Our study did not find a significant association between a history of miscarriage or the presence of chronic diseases and depressive or anxiety symptoms. However, previous research has shown that women with a history of miscarriage often report higher levels of psychological distress, including anxiety and depressive symptoms, during subsequent pregnancies^47^. Similarly, the presence of chronic conditions has been linked to an increased risk of mental illness in other studies^48^. The lack of observed associations in our study may be attributed to the relatively small sample size or a low number of participants with these specific characteristics, potentially limiting the statistical power to detect such relationships.

The study has several limitations that need to be acknowledged. The study was conducted in a single public health facility in a rural region of Bangladesh, which may restrict the generalizability of findings to other urban and private settings. This study only included participants visiting a healthcare facility for ANC services, potentially missing the other ANC women within the community who did not seek ANC care from a health facility. This limitation could lead to overestimation or underestimation of the findings. While PHQ-9 and GAD-7 were used, reliance on self-reported data may be subject to reporting bias or social desirability bias, potentially affecting the accuracy of responses. Additionally, the consecutive sampling, though practical, may have introduced selection bias, as women who did not attend the health facility for ANC care during the data collection period or received care from other facilities were not included. Furthermore, the study did not account for the potential confounders such as cultural factors, dietary factors, and partner supports, which could influence mental health outcomes. In particular, broader societal, cultural, and religious influences such as gender norms, community stigma around mental illness, religious coping practices, and social expectations during pregnancy may play a significant role in shaping mental health during the antenatal period. Finally, the cross-sectional design of the study limits the ability to draw a causal relation between anxiety and depressive symptoms, as it was collected at a single point in time.

## Conclusion

Our study highlights a significant prevalence of depressive and anxiety symptoms among antenatal care seekers, with notable co-occurrence of these conditions. While severe symptoms were not observed, moderate levels of fatigue, nervousness, and fear were prevalent. The findings suggest a dynamic relationship between trimester, education, and mental health, with symptoms generally decreasing as pregnancy progresses and higher education levels associated with a reduced likelihood of mental health issues.

The findings underscore the urgent need for mental health support for women seeking antenatal care in healthcare facilities, with a particular emphasis on early pregnancy, a period marked by heightened vulnerability to mental health challenges. Interventions during this stage can address prevalent symptoms such as fatigue, nervousness, and fear, reducing their impact on maternal well-being. Globally, these findings point to the need for further research on the intricate relationship between maternal mental health and antenatal care to deepen our understanding of how these challenges manifest across diverse cultural and socioeconomic contexts. In Bangladesh, this study highlights the urgent need to incorporate health facility-based tele-mental healthcare services into the broader maternal health frameworks to address the acute shortage of mental health professionals and limited healthcare resources. This integration should target antenatal care (ANC) seekers, particularly those in rural and underserved areas, where access to mental health support is critically low. Healthcare policymakers, in collaboration with local and international organizations, should design scalable and sustainable telehealth platforms that provide mental health screening, virtual counseling, and psychoeducation tailored to the unique challenges of pregnant women.

## Electronic supplementary material

Below is the link to the electronic supplementary material.

Supplementary Material 1

## Data Availability

The anonymised datasets utilised in the present study are not accessible to the public as a precautionary measure to safeguard the confidentiality of participants. However, interested researchers may obtain access to these datasets upon making a reasonable request to the corresponding author.

## Abbreviations

ANC: Antenatal Care
CI: Confidence Interval
DGHS: Director General of Health Services
DH: District Hospital
DSM: Diagnostic and Statistical Manual of Mental Disorders
GAD-7: Generalized Anxiety Disorder-7
icddr,b: International Centre for Diarrhoeal Disease Research, Bangladesh
IRB: Institutional Review Board
LMIC: Low- and Middle-Income Countries
MoHFW: Ministry of Health and Family Welfare
NCDC: Non-communicable Disease Control
NIMH: National Institute of Mental Health
OR: Odds Ratio
PHQ-9: Patient Health Questionnaire-9
UHC: Upazila Health Complex
WHO: World Health Organization
