# Supplementary material for: Depressive, anxiety symptoms and their co-occurrence among women seeking antenatal care in Bangladesh
Source: Sci Rep. 2025 May 17;15:17137. doi: 10.1038/s41598-025-01801-w (PMC12085565; doi:10.1038/s41598-025-01801-w)
Supplement: Supplementary file 1 — Supplementary Material 1 [file 41598_2025_1801_MOESM1_ESM.docx]

Supplementary material 1: Map of the study area

The map presented in Supplementary Figure 1 was created by a co-author from our team, NGU, using ArcGIS software (version 10.8, ESRI, https://www.esri.com/en-us/arcgis/about-arcgis/overview). No copyrighted material was used or modified in its creation.


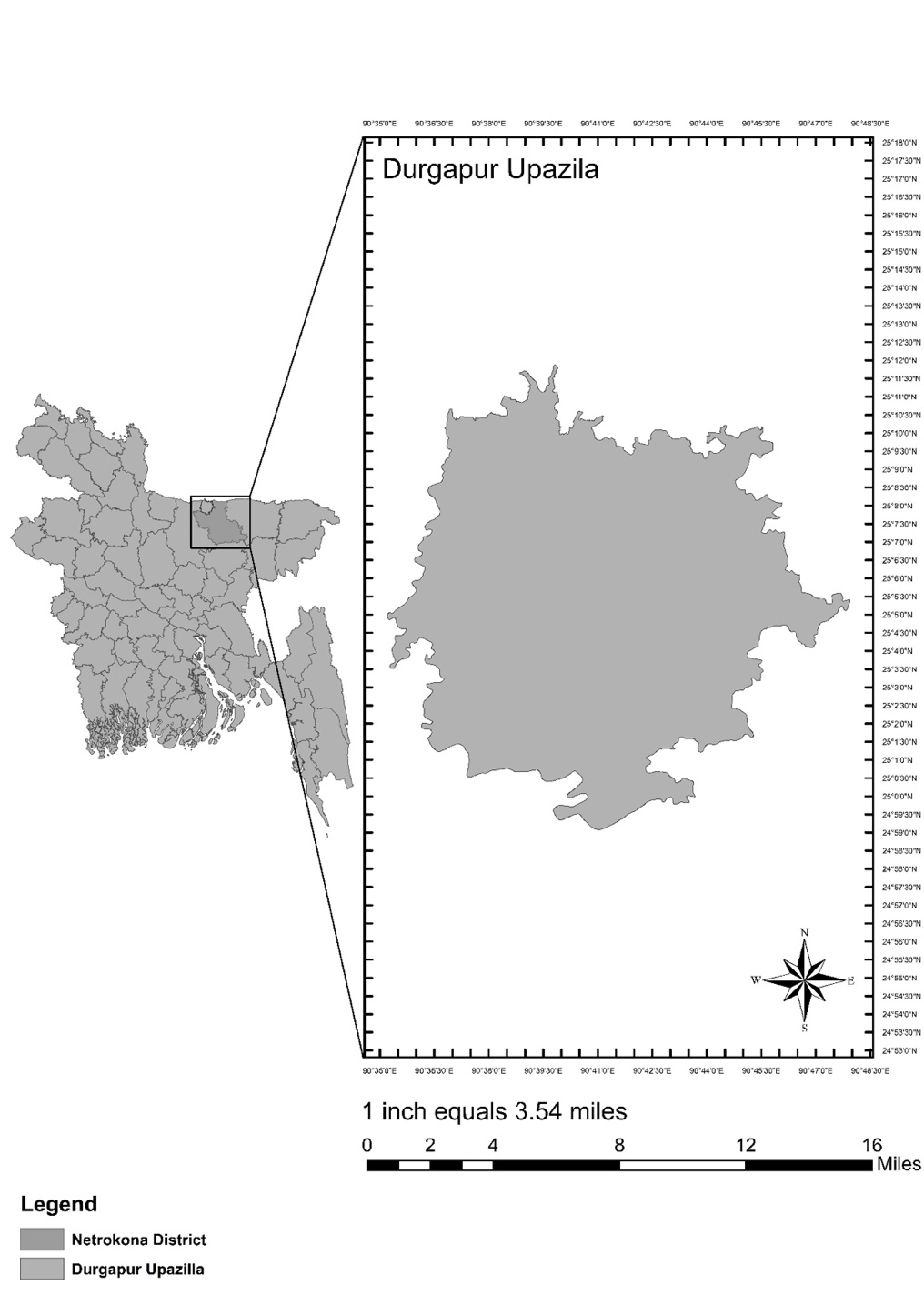


Supplementary material 2: The socio-demographic and socio-economic variables used in the study

- Age in years (The length of time an individual has lived, calculated from their date of birth to the date of data collection, measured in full years.)

We categorized the variable into 17–20 years, 21–25 years, and 26–40 years based on commonly used age groupings in maternal health research, which align with developmental and reproductive health stages, as well as to facilitate meaningful comparisons with previous studies in similar contexts.

- Years of education (The total number of years an individual has spent receiving formal education in a structured setting, such as schools, colleges, or universities.)

The study categorized years of education into 0–5 years, 6–10 years, and ≥11 years based on common educational milestones in Bangladesh, with 0–5 years representing primary education, 6–10 years reflecting secondary education, and ≥11 years indicating higher secondary or above, to align with national education levels and facilitate meaningful analysis.

- Household income (The total monetary income earned by a household member combined in one month, measured in Bangladeshi taka, BDT).

Household income was classified into <10,000 (Very low), 10,000–19,999 (Lower middle), 20,000–29,999 (Middle), and ≥30,000 (High) based on standard income brackets commonly used in socioeconomic research in Bangladesh, reflecting varying levels of economic stability and purchasing power.

- Gestational weeks were grouped into 1–12 weeks, 13–28 weeks, and 29–40 weeks to correspond with the three trimesters of pregnancy, which are standard stages used in obstetric research and clinical practice to assess and monitor maternal and fetal development.
- Total number of living children (The total count of biological offspring of an individual who are alive at the time of data collection.)
  The study recorded the number of living children as no children, 1, or ≥2.
- First pregnancy (Pregnancy is the term used to describe the period in which a fetus develops inside a woman's womb or uterus and a women experiencing for the first time.)
  Participants were identified as being in their first pregnancy (yes or no).
- Mode of last delivery (The method used to deliver a baby in the most recent childbirth event, including natural (vaginal), surgical (C-section), or terminated (abortion).)
  The mode of the last delivery was noted as normal, C-section, or abortion.
- History of miscarriage (A miscarriage is the loss of a pregnancy during the first 23 weeks.)
  The study documented whether participants had a history of miscarriage (yes or no).
- History of dead child (An account of a child born alive to a participant who subsequently died at any point before the study.)
  Information on a history of a dead child was recorded (yes or no).
- Suffering from chronic disease for 12 months or more. (Chronic diseases are defined broadly as conditions that last 1 year or more and require ongoing medical attention or limit activities of daily living or both.)
  Chronic diseases lasting 12 months or more were recorded as yes or no. The follow-up question recorded the number of chronic disease present at the moment of data collection in the participant. The study noted the total number of chronic diseases as 1 or ≥2.

Supplementary material 3: Variance Inflation Factors (VIF).

| **Constructs** | **VIF** |
| --- | --- |
| Mode of last delivery | 1.99 |
| Total living children | 1.67 |
| Miscarriage history | 1.42 |
| Total number of chronic diseases | 1.22 |
| Age, years | 1.20 |
| Household income, taka per month | 1.19 |
| Gestational period | 1.13 |
| Education, years | 1.13 |
| History of child death | 1.06 |

Supplementary material 4: List of models excluded

**A. Depressive Symptoms**

| **Model ID** | **Covariates Included** | **Hosmer–Lemeshow p-value** |
| --- | --- | --- |
| D1 (Full Model) | Age, education, income, gestational period, total children, first pregnancy, miscarriage history, chronic disease, child death history | 0.018 *(excluded)* |
| D2 | Removed first pregnancy and child death history | 0.041 *(excluded)* |
| D3 | Removed age and income | 0.060 *(excluded – borderline fit but unstable estimates)* |
| D4 (Final Model) | Education, gestational period, total children, miscarriage history, chronic disease | 0.684 *(included)* |

**B. Anxiety Symptoms**

| **Model ID** | **Covariates Included** | **Hosmer–Lemeshow p-value** |
| --- | --- | --- |
| A1 (Full Model) | Age, education, income, gestational period, total children, first pregnancy, miscarriage history, chronic disease, child death history | 0.012 *(excluded)* |
| A2 | Removed income and child death history | 0.039 *(excluded)* |
| A3 | Removed age and first pregnancy | 0.058 *(excluded)* |
| A4 (Final Model) | Education, total children, miscarriage history, chronic disease | 0.611 *(included)* |

**C. Co-occurrence of Depression and Anxiety**

| **Model ID** | **Covariates Included** | **Hosmer–Lemeshow p-value** |
| --- | --- | --- |
| C1 (Full Model) | Age, education, income, gestational period, total children, first pregnancy, miscarriage history, chronic disease, child death history | 0.015 *(excluded)* |
| C2 | Removed income and child death history | 0.036 *(excluded)* |
| C3 | Removed age and first pregnancy | 0.070 *(borderline fit, unstable estimates)* |
| C4 (Final Model) | Education, gestational period, total children, miscarriage history, chronic disease | 0.732 *(included)* |
